# Leveraging the influenza sentinel surveillance platform for SARS-CoV-2 monitoring in Bangladesh (2020–2024): a prospective sentinel surveillance study

**DOI:** 10.1016/j.lansea.2025.100657

**Published:** 2025-08-21

**Authors:** Md Ariful Islam, Md Zakiul Hassan, Zubair Akhtar, Saju Bhuiya, Tanzir Ahmed Shuvo, Probir Kumar Ghosh, Asadullah, Md Abdullah Al Jubayer Biswas, Mustafizur Rahman, Mohammad Jubair, Mst Noorjahan Begum, Yeasir Karim, Mohammed Ziaur Rahman, Mohammad Enayet Hossain, Mohammad Niaz Morshed Khan, Tahmina Shirin, Shah Niaz Md Rubaid Anwar, Ahmed Nawsher Alam, Mohammad Ferdous Rahman Sarker, Monalisa, Manjur Hossain Khan Jony, Mahbubur Rahman, Mahmudur Rahman, Mohammad Abdul Aleem, Fahmida Chowdhury

**Affiliations:** a International Centre for Diarrhoeal Disease Research, Bangladesh (icddr,b), Dhaka, 1212, Bangladesh; b School of Population Health, University of New South Wales (UNSW), Sydney, NSW, 2052, Australia; c Department of Medicine, University of Oxford, Oxford, UK; d Biosecurity Program, Kirby Institute, University of New South Wales (UNSW), Sydney, NSW, 2052, Australia; e Institute of Epidemiology, Disease Control and Research (IEDCR), Dhaka, 1212, Bangladesh; f Global Health Development/EMPHNET, Dhaka, 1212, Bangladesh

**Keywords:** COVID-19, SARS-CoV-2, Influenza, Monitoring, Integration, Surveillance, Sentinel surveillance, Bangladesh

## Abstract

**Background:**

There is limited global evidence on whether influenza sentinel surveillance platforms can be effectively adapted for long-term SARS-CoV-2 monitoring in low-resource contexts. We explored the utility of the hospital-based influenza sentinel surveillance (HBIS) platform for monitoring SARS-CoV-2 in Bangladesh by comparing SARS-CoV-2 detection in HBIS platform with national COVID-19 platform and assessing how its integration into influenza surveillance aligns with national trends.

**Methods:**

From March 2020 to December 2024, we analysed data from patients with severe acute respiratory infection (SARI) and influenza-like illness (ILI) enrolled in HBIS. Socio-demographic and clinical data were recorded, and nasopharyngeal and oropharyngeal swabs were tested for influenza and SARS-CoV-2 using rRT-PCR. Whole-genome sequencing was performed on a subset of SARS-CoV-2–positive samples. Data from national COVID-19 platform were obtained from the Directorate General of Health Services, Bangladesh, and were compared with HBIS platform data using epidemic curves and Pearson correlation analysis.

**Findings:**

Among 25,366 (SARI: 20,226; ILI: 5140) patients, 13.0% (3310) tested positive for influenza, 6.6% (1680) for SARS-CoV-2, and 0.2% (43) were co-infected. SARS-CoV-2 positivity in HBIS (6.8%), including 0.2% co-infections, was lower than the national average (13.1%), but showed a strong correlation with national trends (Pearson *r* = 0.86, P < 0.001). Sequencing of 234 SARS-CoV-2 strains detected the beta and delta variants in April and May 2021, respectively, and omicron subvariants circulating from 2022 to 2024, aligning with the national COVID-19 platform.

**Interpretation:**

SARS-CoV-2 positivity trends in HBIS platform closely aligned with the national COVID-19 platform, demonstrating its potential as a sustainable platform for COVID-19 monitoring. Our findings underscore the feasibility of influenza sentinel surveillance as an early warning system for future COVID-19 outbreaks or other respiratory viruses of pandemic concern in Bangladesh and similar settings.

**Funding:**

10.13039/100000030Centers for Disease Control and Prevention (CDC), Atlanta, Georgia, USA (U01GH002259).

**Research in context**

**Evidence before this study**

During the early phase of the COVID-19 pandemic, the WHO's Global Influenza Surveillance and Response System recommended that countries integrate SARS-CoV-2 surveillance into their existing influenza sentinel surveillance platforms. We searched PubMed and Google Scholar using a combination of search terms (i.e., “SARS-CoV-2,” “COVID-19,” “monitoring,” “integration,” “influenza surveillance,” “genomic surveillance,” “genomic sequencing”) to identify articles on the integration of SARS-CoV-2 into influenza surveillance. Moreover, we reviewed relevant WHO recommendations and reports on the integrated surveillance of SARS-CoV-2 and influenza. The scope of the review included studies and guidelines published in English from 2020 to 2024. The review revealed that many countries incorporated SARS-CoV-2 surveillance into influenza surveillance platforms, focusing on the feasibility and advantages of integration, with some evidence highlighting the potential for leveraging these systems for SARS-CoV-2 monitoring. However, there is a lack of studies exploring how this integration aligns with national COVID-19 data systems or how influenza sentinel surveillance platforms could serve as a sustainable platform for long-term COVID-19 monitoring.

**Added value of this study**

We demonstrate that SARS-CoV-2 surveillance can be effectively included and managed within an existing influenza sentinel surveillance platform. SARS-CoV-2 was a major cause of both influenza-like illness and severe acute respiratory infection during 2020–2022, while influenza was the predominant cause during 2023–2024 in Bangladesh. By using this sentinel surveillance platform, we were able to identify the SARS-CoV-2 variants circulating in the country. This information is crucial for informing public health interventions, such as vaccination strategies and targeted testing.

**Implications of all the available evidence**

Our study highlights the potential of influenza sentinel surveillance systems as sustainable platforms for monitoring COVID-19 in Bangladesh and similar settings, serving as an early warning system for future outbreaks of COVID-19 or other respiratory viruses with pandemic potential and public health significance.

## Introduction

SARS-CoV-2, a highly transmissible and pathogenic virus, emerged in late 2019, causing the COVID-19 pandemic, which severely impacted human health and public safety and posed significant challenges to health systems worldwide.^1^ Bangladesh reported its first COVID-19 cases on March 8, 2020,^2^ and subsequently experienced five waves of the pandemic.^3^ To control the spread of the virus, the Government of Bangladesh implemented various measures, including health screenings, isolation, lockdowns, travel restrictions, and the temporary closure of educational institutions.^4^^,^^5^ By February 22, 2022, when most restrictions were lifted, Bangladesh had reported 1,950,846 confirmed COVID-19 cases and 29,117 deaths attributed to the virus. By Dec 31, 2024, these numbers had increased to 2,051,547 confirmed COVID-19 cases and 29,499 deaths.^6^^,^^7^

To monitor SARS-CoV-2, the government established event-based, community-based, and hospital-based surveillance systems. However, these measures were often temporary and dependent on the pandemic's status. A sustainable surveillance platform is needed to monitor SARS-CoV-2 in Bangladesh and support decision-making for epidemic and pandemic preparedness. In the early stages of the COVID-19 pandemic, the WHO recommended integrated surveillance by adapting existing respiratory disease surveillance systems to monitor SARS-CoV-2.^8^, ^9^, ^10^ Integrated surveillance involves the systematic screening of individuals, collection of respiratory specimens, diagnostic testing and sequencing, data analysis, and result dissemination to inform public health interventions and policies. In response to WHO's recommendation, many countries adapted their influenza surveillance platforms to monitor SARS-CoV-2 circulation and provide critical information for pandemic preparedness.^10^, ^11^, ^12^, ^13^ In March 2020, we leveraged the hospital-based influenza sentinel surveillance (HBIS) platform in Bangladesh in response to the COVID-19 pandemic. Since then, the HBIS platform has been utilised to monitor both influenza and SARS-CoV-2, with data reported to the Global Influenza Surveillance and Response System (GISRS). Maintaining a dedicated surveillance platform exclusively for SARS-CoV-2 is resource-intensive and labour-demanding, making it potentially unsustainable in resource-constrained settings like Bangladesh. Thus, it is crucial to evaluate whether the HBIS platform provides sufficient data to serve as a viable alternative to a standalone national COVID-19 platform.

In this study, we compare SARS-CoV-2 detection from the HBIS platform with that of the national COVID-19 platform. We describe how SARS-CoV-2 data from influenza sentinel surveillance aligns with national COVID-19 platform's data and assess the feasibility of integrating SARS-CoV-2 monitoring into the influenza sentinel surveillance system for national level monitoring of SARS-CoV-2 in Bangladesh.

## Methods

### Surveillance sites and population

**Fig. 1:**
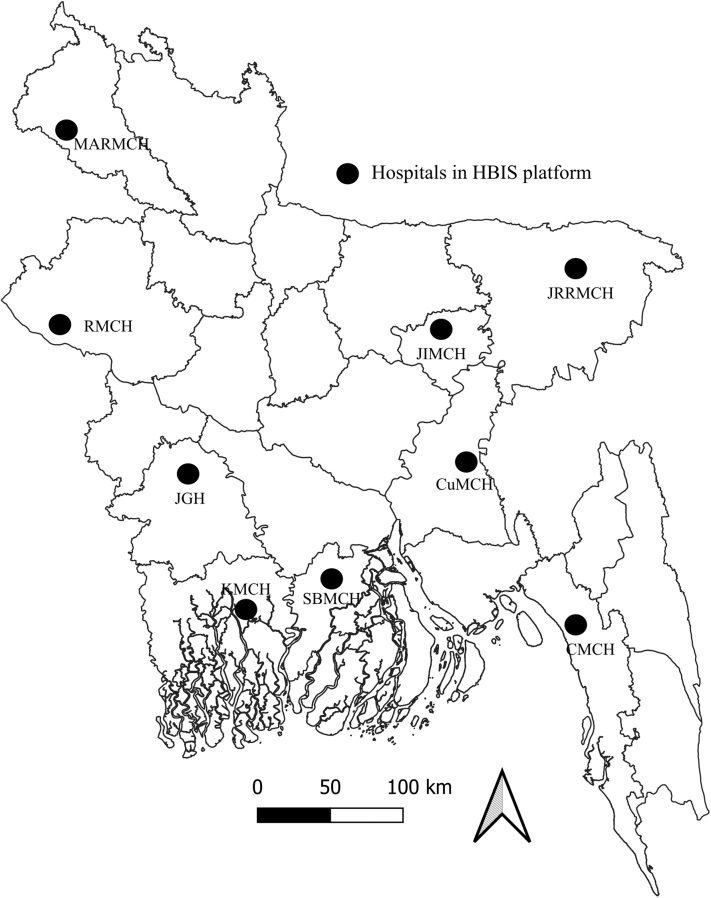
Locations of nine hospitals in the HBIS platform in Bangladesh. 1. Jahurul Islam Medical College Hospital (JIMCH), Kishoregonj; 2. Rajshahi Medical College Hospital (RMCH), Rajshahi; 3. Cumilla Medical College Hospital (CuMCH), Cumilla; 4. Khulna Medical College Hospital (KMCH), Khulna; 5. Jashore 250 bed General Hospital (JGH), Jashore; 6. Jalalabad Ragib-Rabeya Medical College Hospital (JRRMCH), Sylhet; 7. Sher-e-Bangla Medical College Hospital (SBMCH), Barishal; 8. Chattogram Medical College Hospital (CMCH), Chattogram; 9. M Abdur Rahim Medical College Hospital (MARMCH), Dinajpur. HBIS: Hospital-based influenza sentinel surveillance.

The HBIS platform in Bangladesh was established in April 2007 through a collaboration between the Institute of Epidemiology, Disease Control and Research (IEDCR) of the Government of Bangladesh, International Centre for Diarrhoeal Disease Research, Bangladesh (icddr,b), and the US Centers for Disease Control and Prevention (CDC). The surveillance aimed to identify individuals and clusters with life-threatening influenza infections and characterise the diversity of circulating strains in Bangladesh. In March 2020, in response to the COVID-19 pandemic, and in accordance with WHO recommendations, we leveraged the HBIS platform for COVID-19 case detection. This platform comprises a network of nine tertiary-care hospitals in Bangladesh, including seven public and two private hospitals, strategically selected to ensure broad geographic coverage and to represent both urban and rural populations (Fig. 1). The catchment areas of these hospitals collectively cover an estimated ∼14% of the population of Bangladesh. Details on catchment area definitions of the hospitals, coverage calculation, and representativeness are provided elsewhere.^14^ Surveillance activities were conducted in the inpatient departments of medicine, paediatric wards, and coronary care units (CCU) to enrol patients with severe acute respiratory infections (SARI). In November 2022, surveillance activities expanded to the outpatient departments (OPDs) of medicine and paediatric wards in five selected hospitals to enrol patients with influenza-like illness (ILI) (Fig. 1). As outpatient services and general admissions in all the hospitals were closed one day each week (weekends), enrolment of patients with SARI and ILI remained operational six days a week (weekdays).

### Case identification

Physicians from the sentinel surveillance team regularly visited medicine and paediatric wards, as well as CCUs, to monitor new admissions for individuals meeting the WHO-defined case definition of SARI. According to WHO case definition, SARI was defined as “a patient with subjective or measured fever of ≥38C° and cough with onset within the past 10 days and requiring hospital admission”.^15^^,^^16^ Field assistants from the sentinel surveillance team visited the OPDs of medicine and paediatric wards to identify and enrol patients meeting the WHO-defined case definition of ILI. ILI was defined as “a patient with measured fever of ≥38C° and cough, with onset within the past 10 days”.^15^

### Specimen collection and laboratory investigations

Surveillance physicians, supported by field assistants, collected nasopharyngeal and oropharyngeal swabs from enrolled patients with SARI and ILI after obtaining written informed consent. During the influenza season in Bangladesh (April–September),^17^ all SARI samples were tested for influenza viruses and SARS-CoV-2 using real-time reverse transcription polymerase chain reaction (rRT-PCR). From October to March, every second SARI sample from patients aged ≥5 years and two randomly selected samples from children aged <5 years per surveillance hospital each week were tested for influenza viruses and SARS-CoV-2.^17^ For ILI sample testing, every third patient with ILI at the designated OPDs in each of the five selected hospitals each day (six days a week) underwent testing for influenza viruses and SARS-CoV-2. Beginning in November 2021, the influenza SARS-CoV-2 (Flu SC2) multiplex assay was introduced to simultaneously detect influenza viruses and SARS-CoV-2. Viral nucleic acid (RNA) was extracted and purified from nasopharyngeal swab samples using either an automated extractor (KingFisher Flex96 system) with an Invimag Pathogen kit or manually with a QiaAmp-viral mini kit. The RT-qPCR was carried out following the CDC Flu SC2 Multiplex Assay protocol.^18^

Whole Genome Sequencing of SARS-CoV-2 was performed utilising the Illumina MiSeq and Oxford Nanopore MinION platforms. The sample selection criteria for sequencing was performed based on stringent criteria, specifically targeting samples with cycle threshold (Ct) values of ≤27 to ensure sufficient viral load for high-quality genomic data. This Ct cutoff was chosen to optimise the yield of amplifiable genetic material, minimising the risk of sequencing artifacts or low-coverage regions due to degraded or low-abundance RNA. Sequencing was carried out at virology laboratory and genome centre of icddr,b employing both the Illumina MiSeq and Oxford Nanopore MinION platforms. For Illumina-based sequencing, libraries were prepared using the Illumina COVIDseq™ assay (20051273). Indexed libraries were generated and pooled prior to sequencing. These pooled libraries underwent 300 cycles of sequencing using the MiSeq V3 2 × 300 cycle kit (MS-102-3003).

For samples sequenced on the Oxford Nanopore platform, viral RNA was reverse transcribed into cDNA with the Lunascript RT SuperMix Kit (NEB, E3010). The resulting cDNA was amplified using the ARCTIC protocol to target key genomic regions for surveillance. Sequencing libraries were then prepared with the Native Barcoding Kit (EXP-NBD104) and run on R9 flow cells for 8 h to enable real-time data collection and analysis. Sequencing data were processed using the DRAGEN COVID Lineage and EPI2ME analysis pipelines to generate high-quality genomic assemblies. Most of the resulting sequences attained genome coverage levels between 95% and 100%.

### Data collection

#### Primary data collection from HBIS platform

Between March 2020 and December 2024, sentinel Surveillance physicians collected surveillance data from patients with SARI and ILI using a standardised surveillance case record form. These data included socio-demographic information such as age, sex, and residential status, clinical information (e.g., fever, cough, runny nose, headache, sore throat, difficulty breathing reported on admission), and co-morbid conditions. Moreover, reports of any danger signs among children aged <5 years (i.e., chest indrawing, stridor in a calm child, inability to drink, lethargy or unconsciousness, vomiting everything, or history of convulsions) were recorded, along with laboratory data (blood and chest radiograph findings) (Table 1, Supplementary Table S1). Hospital discharge outcomes (alive or death) of patients with SARI were also documented. All collected data were transferred in real-time to icddr,b's central server. Influenza and SARS-CoV-2 test results were collected from icddr,b's virology laboratory and merged with the respective patient epidemiological information.

**Table 1:** Demographic, clinical and epidemiological characteristics of SARS-CoV-2–infected SARI and ILI patients identified through hospital-based influenza surveillance, Bangladesh, 2020–2024.

Characteristics	SARI enrolled	SARS-CoV-2 positivity in SARI	ILI enrolled	SARS-CoV-2 positivity in ILI	SARI & ILI enrolled	SARS-CoV-2 positivity in SARI & ILI
	N = 20,226	N = 1545	N = 5140	N = 178	N = 25,366	N = 1723
	n (%)	n (%)	n (%)	n (%)	n (%)	n (%)
**Demographic characteristics**						
Age						
0–4 years	8353 (41.3)	211 (13.7)	1103 (21.5)	24 (13.5)	9456 (37.3)	235 (13.6)
5–14 years	2350 (11.62)	44 (2.9)	1019 (19.8)	26 (14.6)	3369 (13.3)	70 (4.1)
15–29 years	2399 (11.86)	173 (11.2)	1642 (32.0)	72 (40.5)	4041 (15.9)	245 (14.2)
30–65 years	5206 (25.74)	826 (53.5)	1239 (24.1)	53 (29.8)	6445 (25.4)	879 (51.0)
65 years and above	1918 (9.48)	291 (18.8)	137 (2.7)	3 (1.7)	2055 (8.1)	294 (17.1)
Median age (IQR), years	11 (0.9–45)	45 (26–60)	18 (6–30)	21 (11–34)	14 (1.2–40)	42 (23–60)
Male	12,750 (63.0)	939 (60.8)	3034 (59.0)	103 (57.9)	15,784 (62.2)	1042 (60.5)
**Clinical characteristics**						
**Patients aged<5 years**	**N** = **8353**	**N** = **211**	**N** = **1103**	**N** = **24**	**N** = **9456**	**N** = **235**
Chest indrawing	4279 (51.2)	114 (54.0)	0 (0)	0 (0)	4279 (51.2)	114 (54.0)
Stridor	813 (9.7)	26 (12.3)	0 (0)	0 (0)	813 (9.7)	40 (24.2)
Unable to drink	2008 (24.0)	53 (25.1)	0 (0)	0 (0)	2008 (24.0)	53 (25.1)
Vomit	471 (5.7)	9 (4.3)	0 (0)	0 (0)	471 (5.7)	9 (4.3)
Lethargy	76 (0.9)	2 (0.9)	0 (0)	0 (0)	76 (0.9)	2 (0.9)
Runny nose	5985 (71.7)	145 (68.7)	900 (81.6)	19 (79.2)	6885 (72.8)	164 (69.8)
**Patients aged ≥5 years**	**N** = **11,873**	**N** = **1334**	**N** = **4037**	**N** = **154**	**N** = **15,910**	**N** = **1488**
Body ache	5988 (50.4)	713 (53.5)	1142 (28.3)	51 (33.3)	7143 (33.7)	764 (51.3)
Headache	5934 (50.0)	628 (47.1)	1554 (38.5)	67 (43.5)	7488 (47.1)	695 (46.7)
Sore throat	1792 (26)	273 (22.6)	740 (100)	41 (100)	2532 (33.2)	314 (25.2)
**All age group**	**N** = **20,226**	**N** = **1545**	**N** = **5140**	**N** = **178**	**N** = **25,366**	**N** = **1723**
Difficulty breathing reported on admission	13,850 (85.0)	1195 (82.8)	606 (100)	24 (100)	14,456 (85.5)	1219 (83.0)
Duration of symptoms prior to admission in days; Median (IQR)	5 (3–6)	5 (4–7)	3 (2–5)	3 (2–4)	4 (3–6)	5 (3–7)
Length of hospital stay in days; Median (IQR)	3 (2–5)	3 (2–6)	0 (0)	0 (0)	3 (2–5)	3 (2–6)
**Comorbidity**	**N = 20,226**	**N = 1545**	**N = 5140**	**N = 154**	**N = 25,366**	**N = 1723**
≥1 co-morbid condition (Self-reported)	4232 (20.9)	580 (37.7)	212 (4.12)	8 (4.5)	4444 (17.5)	588 (34.1)
Chronic obstructive pulmonary disease	542 (2.7)	34 (2.2)	12 (0.2)	0 (0)	554 (2.2)	34 (2.0)
Asthma	1475 (7.3)	132 (8.5)	75 (1.5)	2 (1.1)	1550 (6.1)	134 (7.8)
Diabetes	1400 (6.9)	310 (20.1)	78 (1.5)	2 (1.1)	1478 (5.8)	312 (18.1)
Heart diseases	811 (4.0)	72 (4.7)	44 (0.9)	2 (1.1)	855 (3.4)	74 (4.3)
Hypertension	1806 (8.9)	337 (21.8)	25 (0.5)	4 (2.2)	1831 (7.2)	341 (19.8)
Cancer	23 (0.1)	2 (0.1)	2 (0.04)	1 (0.6)	25 (0.1)	3 (0.2)
Liver disease	32 (0.2)	4 (0.3)	3 (0.1)	0 (0)	35 (0.1)	4 (0.2)
Kidney disease	114 (0.6)	21 (1.4)	6 (0.1)	0 (0)	120 (0.5)	21 (1.2)
**Treatment received**	**N = 20,226**	**N = 1545**	**N = 5140**	**N = 154**	**N = 25,366**	**N = 1723**
Antibiotic	17,901 (88.5)	1457 (94.3)	2582 (50.2)	87 (48.2)	20,483 (80.8)	1544 (89.6)
Oxygen	8298 (41.0)	980 (63.4)	0 (0)	0 (0)	8298 (41.0)	980 (63.4)
Mechanical ventilation	9 (0.04)	2 (0.1)	0 (0)	0 (0)	9 (0.04)	2 (0.1)
ICU support (after admission in the general ward)	17 (0.1)	4 (0.3)	0 (0)	0 (0)	17 (0.1)	4 (0.3)
**Laboratory results**	**N = 20,226**	**N = 1545**	**N = 5140**	**N = 154**	**N = 25,366**	**N = 1723**
SARS-CoV-2 detected	1507 (7.5)	–	173 (3.4)	–	1680 (6.6)	–
Influenza virus detected	2514 (12.4)	–	787 (15.4)	–	3301 (13.0)	–
Co-infection (SARS-CoV-2 and influenza)	38 (0.2)	–	5 (0.3)	–	43 (0.2)	–
**Clinical outcome**	**N = 20,226**	**N = 1545**			**N = 20,226**	**N = 1545**
Death^a^	530 (2.6)	136 (8.8)	–	–	530 (2.6)	136 (8.8)

SARI: severe acute respiratory infection.

ILI: Influenza-like illness.

a Death was identified only among patients with SARI.

### Secondary data collection (data extraction) from the national COVID-19 platform in Bangladesh

Data on SARS-CoV-2 were extracted from the national COVOD-19 platform, which is maintained by the Directorate General of Health Services (DGHS) under the Ministry of Health and Family Welfare (MOHFW), Government of Bangladesh. Publicly available national COVID-19 platform data were downloaded from the DGHS website of the MOHFW for the period between March 2020 and December 2024.^7^ Daily data on the number of SARS-CoV-2 tests conducted, confirmed cases, and positivity rates were compiled into a Microsoft Excel file and aggregated into weekly and monthly formats as needed for analysis. In response to the COVID-19 pandemic, the Government of Bangladesh initiated COVID-19 screening and contact tracing through event-based surveillance in early 2020. Initially, WHO-recommended rRT-PCR-based COVID-19 testing facilities were established at IEDCR.^19^ Subsequently, testing facilities were expanded to selected government and non-government institutions across the country, with provisions to share daily COVID-19 test results with the MOHFW through the DGHS. The SARS-CoV-2 testing facilities, which utilised rRT-PCR, GeneXpert, and rapid antigen tests, were expanded to 885 sites, including 232 rRT-PCR laboratories (162 public and 70 private). As of Dec 31, 2024, approximately 30–40 facilities were actively conducting routine SARS-CoV-2 testing and reporting results to the MOHFW.^6^ The national SARS-CoV-2 case report form was brief, capturing only basic demographic data (age, sex, and residential status) and clinical outcomes (alive or deceased) during the illness episode (Supplementary Table S1). The national COVID-19 platform instructed all testing facilities to perform COVID-19 testing for individuals who met the suspected or probable case definition of SARS-CoV-2, including international travellers requiring a test result for travel purposes. Detailed case definitions for SARS-CoV-2 testing used by the national COVID-19 platform have been published elsewhere.^20^ To facilitate real-time COVID-19 monitoring, all symptomatic and asymptomatic cases undergoing COVID-19 testing were reported to the MOHFW and included in the national COVID-19 platform database. Samples tested for SARS-CoV-2, including those for international travel, were also included in this database. Second, we were unable to conduct detailed comparative analyses between the HBIS platform and the national COVID-19 platform. However, the national COVID-19 platform database did not categorise international travellers separately, making it impossible to exclude them from sensitivity analyses. Notably, SARS-CoV-2 testing results from HBIS platform were not integrated into the national database. Both HBIS and national COVID-19 platform genomic sequencing data were regularly uploaded to Global Initiative on Sharing All Influenza Data (GISAID), a globally accessible database for genomic surveillance.^21^

### Analysis framework for comparing HBIS and the national COVID-19 platform

Our analysis framework systematically compares data from the HBIS platform with the national COVID-19 platform, addressing key priorities for COVID-19 monitoring in Bangladesh. This framework focuses on understanding the circulation patterns (weekly positivity trends) and peak times of SARS-CoV-2, identifying populations at risk of infection (age and sex groups with higher positivity rates), quantifying infection rates and SARS-CoV-2-associated mortality, monitoring weekly SARS-CoV-2 testing numbers, and conducting genomic sequencing to monitor known variants of concern (VOCs) and enable early detection of new VOCs.

### Framework for integrating SARS-CoV-2 into influenza sentinel surveillance (HBIS platform)

We adopted the WHO guidance for integrating SARS-CoV-2 into influenza sentinel surveillance, which has been updated over time, and adapted it accordingly.^8^, ^9^, ^10^^,^^22^^,^^23^ For this analysis, we applied the modified WHO guideline for integrating SARS-CoV-2 into HBIS platform. Key aspects of this integration include (1) incorporating both influenza and SARS-CoV-2 at all stages of the influenza sentinel surveillance process, (2) maintaining quality, representativeness, sustainability, and national ownership throughout the integration process, (3) ensuring that SARS-CoV-2 testing is embedded without compromising the quality of influenza surveillance activities, (4) applying WHO-recommended case definitions for SARI and ILI to maintain standardisation, (5) conducting routine testing of at least 50 specimens per week, with an optimal target of 150, using multiplex rRT-PCR assays, (6) implementing year-round surveillance to ensure continuous assessment of respiratory pathogens, and (7) facilitating the timely upload of SARS-CoV-2 genetic sequence data to publicly accessible platforms such as GISAID, enabling genomic surveillance and early detection of emerging variants.

### Statistical analysis

We analysed weekly epidemiological and virological data on influenza and SARS-CoV-2 from patients with SARI and ILI enrolled under HBIS platform and the national COVID-19 platform of the Government of Bangladesh. Descriptive analyses were conducted to summarise the characteristics of influenza virus- and SARS-CoV-2-positive patients with SARI and ILI identified through the HBIS platform. Weekly SARS-CoV-2 test positivity rates from HBIS platform were compared with national COVID-19 platform test positivity rates reported by the Government of Bangladesh, using frequencies and percentages. Epidemiological curves illustrating the weekly SARS-CoV-2 positivity proportions from HBIS platform were plotted and compared with that of national COVID-19 platform data using Microsoft Excel (Microsoft Corp) charts. Pearson correlation coefficient (*r*) was calculated to assess the correlation between SARS-CoV-2 positivity rates in the HBIS platform and the National COVID-19 platform in Bangladesh. Differences in weekly SARS-CoV-2 positivity proportions between the two platforms were calculated with 95% confidence intervals (CIs). Based on expert consensus, an absolute difference of ≥10 percentage points between the platforms was considered epidemiologically meaningful. Data management and statistical analyses were conducted using Stata 13.0 software (StataCorp. 2013. Stata Statistical Software: Release 15. College Station, TX: StataCorp LP).

### Ethical review

The HBIS protocol was approved by the Institutional Review Board of icddr,b (protocol #2007-002). Participants or legal guardians of children aged <18 years provided written, informed consent before enrolment.

### Role of the funding source

This study was funded by the US Centers for Disease Control and Prevention (CDC). CDC staff contributed to the refinement of the study design, data interpretation, and the development of the manuscript.

## Results

### Study patient characteristics

From March 2020 to December 2024, we approached 25,494 patients meeting SARI or ILI case definitions for enrolment, of these, 0.5% (117) refused to participate, and 25,366 provided written informed consent and were subsequently enrolled in the HBIS platform. Among these 79.7% (20,226) were patients with SARI, and 20.3% (5140) were patients with ILI. The median age of the enrolled patients was 14 years (interquartile range [IQR]: 1.2–40), and 62.2% were male. The median weekly enrolment was 77 patients (IQR: 49–118, range: 1–249 over six workdays). Among the patients with SARI, the median duration from symptom onset to hospitalisation was 4 days (IQR: 3–6), and the median length of hospital stay was 3 days (IQR: 2–5). The median time from sample collection to laboratory result availability was 8 days (IQR: 5–10). Of all enrolled patients, 6.6% (1680/25,366) tested positive for SARS-CoV-2, 13.0% (3301/25,366) tested positive for influenza virus, and 0.2% (43/25,366) had coinfections with both viruses (Tables 1 and 2).

**Table 2:** Annual influenza and SARS-CoV-2 positivity from the HBIS platform and national COVID-19 platform in Bangladesh, 2020–2024.

Year	HBIS platform					National COVID-19 platform	
	Samples tested	Influenza (%)	SARS-CoV-2 (%)	Co-infected with influenza and SARS-CoV-2 (%)	Overall SARS-CoV-2^a^ (%)	Samples tested	SARS-CoV-2 (%)
2020 (March–December)	1994	170 (8.5)	280 (14.0)	5 (0.3)	285 (14.3)	3,239,701	514,500 (15.9)
2021	4431	426 (9.6)	741 (16.7)	5 (0.1)	746 (16.8)	8,267,001	1,071,409 (13.0)
2022	4747	262 (5.5)	279 (5.9)	9 (0.2)	288 (6.1)	3,666,091	451,233 (12.3)
2023	6921	1248 (18.0)	163 (2.4)	7 (0.1)	170 (2.5)	458,659	9170 (2.0)
2024	7273	1195 (16.4)	217 (3.0)	17 (0.2)	234 (3.2)	92,365	5235 (5.7)
**Total**	**25,366**	**3301 (13.0)**	**1680 (6.6)**	**43 (0.2)**	**1723 (6.8)**	**15,723,817**	**2,051,547 (13.1)**

SARI: Severe acute respiratory infection.

ILI: Influenza-like illness.

HBIS: Hospital-based influenza sentinel surveillance.

a Overall SARS-CoV-2 infection, including co-infections with influenza.

### Clinical characteristics of the patients with SARI and ILI identified from HBIS

Among the enrolled patients (n = 25,366), all presented with fever and cough, as these were mandatory criteria for enrolment. In addition to these symptoms, the most frequently reported clinical manifestations included difficulty breathing (85.5%, 14,456). A total of 17.5% (4444) of patients reported at least one comorbid condition. The most reported comorbidity was hypertension (7.2%, 1831), followed by asthma (6.1%, 1550), diabetes (5.8%, 1478), heart disease (3.4%, 855), and chronic obstructive pulmonary disease (COPD) (2.2%, 554) (Table 1). Patients with SARI had a median age of 11 years (IQR: 0.9–45), with 63.0% male, while patients with ILI were older (median age: 18 years; IQR: 6–30), with 59.0% male. Respiratory difficulty was recorded in 85.0% of patients with SARI and in all patients with ILI. **In children aged <5 years**, chest indrawing (51.2%, 4279) and runny nose (71.7%, 5985) were common among patients with SARI, whereas patients with ILI in this age group predominantly presented with runny nose (81.6%, 900). **In patients aged ≥5 years**, body ache (50.4%, 5988) and headache (50.0%, 5934) were more frequently reported among patients with SARI than among patients with ILI (28.3%, 1142 and 38.5%, 1554, respectively). Sore throat was universally reported among patients with ILI (100.0%, 740) but was less common among patients with SARI (26.0%, 1792). Comorbidities were more frequent among patients with SARI (20.9%, 4232) compared to patients with ILI (4.1%, 212). Antibiotics were administered to 88.5% (17,901) of patients with SARI and 50.2% (2582) of patients with ILI. Oxygen support was required in 41.0% (8298) of patients with SARI. SARS-CoV-2 was detected in 7.5% (1507) of patients with SARI and 3.4% (173) of patients with ILI. Influenza viruses were identified in 12.4% (2514) of patients with SARI and 15.4% (787) of patients with ILI. Co-infections with both SARS-CoV-2 and influenza viruses were detected in 0.2% (38) of patients with SARI and 0.3% (5) of patients with ILI (Table 1).

### Clinical characteristics of the COVID-19 patients identified from HBIS platform

Among the 1723 patients who tested positive for SARS-CoV-2, including 43 with coinfections with influenza viruses, the median age was 42 years (IQR: 23–60), and 60.5% were male. The most reported clinical symptom across all age groups was difficulty breathing (83.0%, 1219). Among patients aged<5 years, the most common signs of clinical severity, apart from difficulty breathing, were chest indrawing (54.0%, 114), followed by an inability to drink (25.1%, 53), stridor (24.2%, 40), vomiting (4.3%, 9), and lethargy (0.9%, 2). Among patients aged ≥5 years, the most reported symptoms were, body ache (51.3%, 764), headache (46.7%, 695), and sore throat (25.2%, 314). Across all age groups, 34.1% (588) of patients were reported to have at least one comorbid condition. Among hospitalised patients, the median duration of symptoms before hospital admission was 5 days (IQR: 3–7), and the median length of hospital stay was 3 days (IQR: 2–6). Oxygen therapy was administered to 63.4% (980) of patients during hospitalisation. Mortality was recorded in 8.8% (136/1545) of hospitalised SARS-CoV-2-positive SARI patients. Among all SARS-CoV-2-positive patients, 89.6% (1544) received at least one antibiotic (Table 1).

### Clinical characteristics and outcomes of patients with SARS-CoV-2, influenza, and co-infections of SARS-CoV-2 and influenza in HBIS platform

SARS-CoV-2–positive patients were older (median age: 42 years; IQR: 23.5–60) compared to those with influenza (16 years; IQR: 3.5–35) and those co-infected with both SARS-CoV-2 and influenza (26 years; IQR: 9–55) (p < 0.001). The proportion of male patients was similar across groups, with males comprising 60.4% of SARS-CoV-2 cases, 58.3% of influenza cases, and 65.1% of co-infected cases (p = 0.259). Fever and cough were reported in all patients, as these were inclusion criteria for enrolment. However, difficulty breathing at enrolment was more frequently reported among co-infected patients (87.5%) and SARS-CoV-2 patients (82.6%) compared to influenza patients (76.9%) (p = 0.002). The proportion of patients with at least one comorbid condition was significantly higher among those with SARS-CoV-2 (34.4%) than among patients with influenza (13.8%) or co-infection (23.3%) (p < 0.001). In-hospital mortality was significantly higher among SARS-CoV-2 patients (9.0%) compared to influenza patients (0.95%), while no deaths were reported among co-infected patients (p < 0.001) (Supplementary Table S2).

### Trends in SARS-CoV-2 detection over time in the HBIS platform

**Fig. 2:**
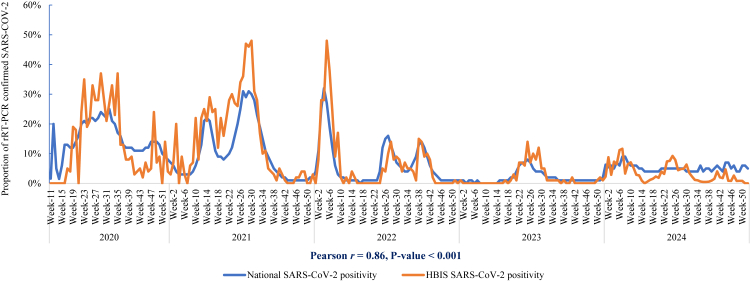
SARS-CoV-2 weekly positivity rate from hospital-based influenza sentinel surveillance platform compared with national COVID-19 platform in Bangladesh, March 2020–December 2024.

**Fig. 3:**
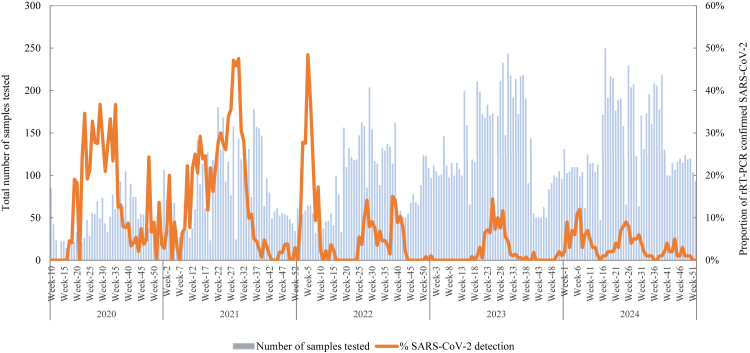
Weekly enrolment of patients with severe acute respiratory infection and influenza-like illness, and SARS-CoV-2 positivity rate through hospital-based influenza sentinel surveillance, Bangladesh, March 2020–December 2024.

The first case of SARS-CoV-2 within HBIS surveillance system was detected during epidemiological (epi) week 17 of 2020 (April 23, 2020). The overall positivity rate for SARS-CoV-2 was 6.8%, of which 6.6% were infected with SARS-CoV-2 alone and 0.2% were co-infections with influenza. The positivity rate varied over time, with annual rates of 14.3% in 2020, 16.8% in 2021, 6.1% in 2022, 2.5% in 2023, and 3.2% in 2024 (Table 2). In 2020, SARS-CoV-2 activity peaked from epi weeks 22–35 (last week of May to the last week of August), with SARS-CoV-2 positivity rates ranging from 19.1% to 36.7%. In 2021, peak activity was observed from epi weeks 26–30 (last week of June to the last week of July), with SARS-CoV-2 positivity ranging from 34.0% to 48.0%. In 2022, the peak period occurred from epi week 3 to epi week 7 (third week of January to the third week of February), with positivity rates ranging from 23.6% to 48.4%. In 2023, SARS-CoV-2 activity peaked from epi week 24 to epi week 29 (second week of June to the third week of July), with positivity rates ranging from 7.0% to 17.0%. In 2024, peak activity was recorded from epi week 6 to epi week 7 (second week of February to the third week of February), with positivity rates ranging from 11.0% to 12.0% (Fig. 2, Fig. 3).

### SARS-CoV-2 variants identified through HBIS platform

**Fig. 4:**
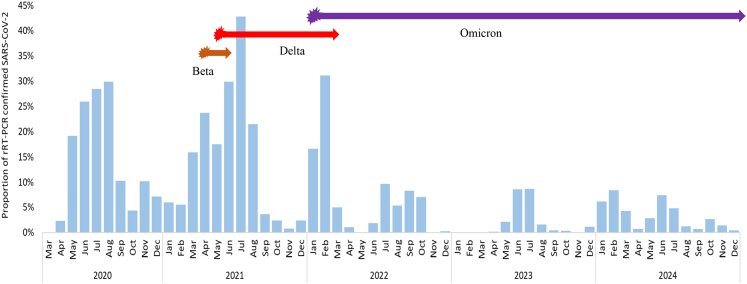
Monthly SARS-CoV-2 positivity and distribution of SARS-CoV-2 variants (beta, delta, omicron) identified through hospital-based influenza sentinel surveillance in Bangladesh, March 2020–December 2024.

Between April 2021 and December 2024, we conducted whole-genome sequencing on 234 SARS-CoV-2 strains, identifying the predominant variants circulating globally during this period. The beta variant was detected in April 2021, followed by the delta variant in May 2021. Omicron sub-variants, including BA.1, BA.2, BA.5, and XBB, were identified through whole-genome sequencing between 2022 and 2024 (Fig. 4). These findings closely align with the national timeline of variant emergence in Bangladesh, where the beta variant was first reported in March 2021, the delta variant in May 2021, and omicron sub-variants (BA.1, BA.2, BA.5, and XBB) between 2022 and 2024.^21^^,^^24^

### SARS-CoV-2 positivity in HBIS platform versus national COVID-19 platform in Bangladesh

The government of Bangladesh reported its first three cases of COVID-19 on March 8, 2020. As of December 2024, a total of 2,051,547 confirmed COVID-19 cases had been reported nationwide, based on 15,723,817 tests, with a weekly average of 62,645 tests (range: 104–334,206). The overall positivity rate in national COVID-19 platform was 13.1% (2,051,547/15,723,817),^6^^,^^7^ including all symptomatic and asymptomatic cases tested, as well as individuals screened for international travel. Including co-infections with influenza, the SARS-CoV-2 positivity rate in the HBIS platform was lower than in the National COVID-19 platform in 2020 (14.3% vs. 15.9%) and 2022 (6.1% vs. 12.3%), but higher in 2021 (16.8% vs. 13.0%) and 2023 (2.5% vs. 2.0%), whereas in 2024, the National COVID-19 platform recorded a higher positivity rate (5.7% vs. 3.2%) (Table 2). The SARS-CoV-2 positivity rate from the HBIS platform, which included 0.2% co-infections, was lower than the national average (6.8% vs. 13.1%; p < 0.001). Overall, the SARS-CoV-2 positivity in HBIS platform was strongly correlated with national COVID-19 platform in Bangladesh (Pearson *r* = 0.86; p < 0.001), with peaks observed during epi weeks 22–35 in 2020, epi weeks 26–30 in 2021, epi weeks 3–7 in 2022, epi weeks 24–29 in 2023, and epi weeks 6–7 in 2024 (Fig. 2). However, statistically significant differences in SARS-CoV-2 positivity between HBIS platform and national COVID-19 platform were observed in 10 epi weeks in 2020 (epi weeks 12, 16, 21, 23, 26, 29, 33, 35, 48, and 51; p < 0.05), 11 epi weeks in 2021 (epi weeks 3, 10, 17, 19, 21, 22, 23, 24, 28, 29, and 30; p < 0.05), and 5 epi weeks in 2022 (epi weeks 5, 6, 7, 9, and 26; p < 0.05), whereas no statistically significant differences were observed in any epi weeks in 2023 and 2024 (Fig. 2, Supplementary Table S3).

### SARS-CoV-2 infected mortality in HBIS platform and the national COVID-19 platform mortality in Bangladesh

The HBIS platform, which solely collected fatal outcome data from patients with SARI and not from those with ILI, identified a SARS-CoV-2 infected SARI death rate of 8.8% (136/1545; 95% CI: 7.4%–10.3%). The national COVID-19 platform in Bangladesh, which included both inpatients and outpatients, symptomatic and asymptomatic cases, as well as international travellers, reported a SARS-CoV-2 infected death rate of 1.4% (29,499/2,051,547; 95% CI: 1.4%–1.5%), as of December 31, 2024.^25^

### Costs associated with SARS-CoV-2 monitoring within the HBIS platform

The integration of SARS-CoV-2 testing into this sentinel surveillance platform incurred minimal additional costs. Staff salaries, operational expenses including costs related to sample collection, transportation, storage, and influenza testing were supported by the HBIS platform. During the initial phase (March 2020 to October 2021), SARS-CoV-2 testing was conducted separately from influenza testing using rRT-PCR, resulting in an incremental cost of approximately US$ 30 per test (around 3653 Bangladeshi Taka [as of Aug 2, 2025]), although actual costs varied depending on the number of samples tested per batch. Since the same respiratory samples collected for influenza testing were used for SARS-CoV-2 testing, no additional costs were incurred for sample collection, logistics, transportation, or storage. In November 2021, the CDC Flu SC2 multiplex assay was introduced into the HBIS platform, enabling simultaneous detection of influenza viruses and SARS-CoV-2 using a single rRT-PCR test. This eliminated the need for separate SARS-CoV-2 testing and did not incur any additional laboratory cost beyond the standard per-sample testing cost (∼USD 30), which was covered by HBIS. Performing whole-genome sequencing of SARS-CoV-2 incurred additional costs, as it was not part of routine HBIS activities. The cost of sequencing was approximately US$ 80 per sample (around 9740 Bangladeshi Taka [as of Aug 2, 2025]), and only a subset of SARS-CoV-2–positive samples (13.6%, 234/1723) underwent sequencing. In contrast, the national COVID-19 platform compiled day-to-day SARS-CoV-2 test results from public and private laboratories across Bangladesh but did not directly conduct or fund laboratory testing. A detailed breakdown of the incremental costs associated with SARS-CoV-2 integration into HBIS is presented in Supplementary Table S4.

### Sharing of SARS-CoV-2 and influenza results from HBIS platform

We maintained year-round influenza sentinel surveillance in Bangladesh, ensuring continuity in monitoring and reporting even during lockdown periods. Weekly test results for both influenza and SARS-CoV-2 were shared with the MoHFW, Government of Bangladesh, and regularly uploaded to the WHO's GISRS platform. In addition, monthly summaries of test results were routinely published on the websites of IEDCR and icddr,b. Genetic sequence data for SARS-CoV-2 were submitted to GISAID. Of the 234 genome sequences generated, 204 were submitted to GISAID, with the corresponding accession identifiers provided in the Supplementary Table S5. The remaining 30 samples underwent targeted Sanger sequencing at the 76_right genomic position and were therefore not deposited in the GISAID database. In Addition, we regularly provided candidate influenza viruses to the WHO to support vaccine composition, production, and risk assessment efforts.

## Discussion

Our study demonstrates that the influenza sentinel surveillance platform effectively captured SARS-CoV-2 circulation patterns in Bangladesh between 2020 and 2024. Temporal patterns of positivity observed in HBIS platform closely aligned with national COVID-19 platform trends in Bangladesh (Pearson's *r* = 0.86; p < 0.001).^7^ Although overall SARS-CoV-2 positivity was lower in HBIS platform (6.8%) compared to the national COVID-19 platform average (13.1%), the temporal distribution of peaks closely mirrored national trends across all major periods of increased transmission during the pandemic. Genomic sequencing from HBIS platform further identified major variants of concern, including beta, delta, and omicron subvariants, which matched the variant profiles reported by the national COVID-19 platform.^21^^,^^24^ These findings underscore the feasibility of integrating SARS-CoV-2 monitoring into existing influenza surveillance infrastructure and highlight its potential as a sustainable approach to respiratory virus surveillance in resource-limited settings.

Our findings, observing close alignment between SARS-CoV-2 trends from influenza sentinel surveillance and the national COVID-19 platform, are consistent with reports from other countries. For instance, findings from ILI and SARI sentinel sites in Indonesia revealed that the overall COVID-19 positivity trend from the sentinel sites aligned with the country's national COVID-19 platform data.^11^ Likewise, an analysis of Kenya's influenza sentinel surveillance data showed a significant correlation with the country's national COVID-19 platform.^26^ These findings underscore the value of influenza sentinel surveillance systems as reliable and sustainable platforms for monitoring SARS-CoV-2 circulation, especially in settings where integrated respiratory surveillance is critical for pandemic preparedness.

Our study found that while the overall SARS-CoV-2 positivity rate detected in HBIS platform was lower than the national COVID-19 platform's average in Bangladesh (6.8% vs. 13.1%), the rates varied across different years. In 2020 and 2022, the HBIS platform reported lower positivity rates compared to the national COVID-19 platform (14.3% vs. 15.9% in 2020 and 6.1% vs. 12.3% in 2022). In contrast, in 2021 and 2023, the HBIS platform recorded higher positivity rates (16.8% vs. 13.0% in 2021 and 2.5% vs. 2.0% in 2023). By 2024, the national COVID-19 platform again reported a higher positivity rate (5.7% vs. 3.2%). Moreover, statistically significant differences in SARS-CoV-2 positivity between the two platforms were observed in 10 epi weeks in 2020, 11 weeks in 2021, and 5 weeks in 2022, whereas no such differences were observed in any epi weeks in 2023 or 2024. These variations likely reflect differences in COVID-19 case definitions, testing coverage and testing criteria across the two platforms, as well as the comparatively low patient enrolment in the HBIS platform during strict lockdown periods. Unlike the HBIS platform, the national COVID-19 platform included a broader population, encompassing asymptomatic individuals, mild cases, travellers, and contacts of confirmed cases, which likely contributed to differences in positivity rates. Moreover, the national COVID-19 platform had a wider network of testing sites, including rapid antigen testing for community-level detection, which may have further influenced overall positivity trends. These findings highlight how variations in surveillance scope and testing strategies can substantially influence positivity rates, emphasising the need for cautious interpretation when comparing data across platforms and for data-driven public health responses.

Our study revealed that the SARS-CoV-2–associated mortality rate was substantially higher among patients with SARI in the HBIS platform (8.8%) compared to the overall mortality rate reported by the national COVID-19 platform (1.4%). This difference likely reflects variations in the populations targeted by each surveillance system. The HBIS platform primarily enrolled patients with SARI, who typically present with more severe illness and are therefore at a higher risk of death, which may have contributed to the elevated mortality rate. Although HBIS included a small number of patients with ILI, but it did not record whether they survived or died during their illness episode. In contrast, the national platform encompassed a much broader spectrum of individuals—including inpatients, outpatients, symptomatic and asymptomatic cases, and international travellers—resulting in a lower overall mortality rate due to the inclusion of milder cases.^20^ These findings underscore the importance of considering case severity and population characteristics when interpreting mortality data across surveillance systems and highlight the potential for methodological biases that may affect reported outcomes.

Even though the WHO lifted its designation of COVID-19 as a “Public Health Emergency of International Concern” (PHEIC), the virus remains a significant global health threat.^27^ Following pandemics, virus mutation rates typically slow down^28^; however, it remains essential to monitor individuals at risk of SARS-CoV-2 infection and to track key factors such as transmissibility, virulence, immune evasion, seasonality, and circulating variants, which may continue to evolve. Given this ongoing threat, it is crucial to maintain influenza sentinel surveillance systems while integrating SARS-CoV-2 testing, as recommended by WHO.^10^^,^^22^ This approach will enable more effective monitoring of both influenza and SARS-CoV-2 circulation, providing valuable data for public health decision-making. By continuing to track these respiratory viruses, we can better prepare for future outbreaks and pandemics. Furthermore, integrating SARS-CoV-2 testing into influenza sentinel systems is especially valuable in regions with limited standalone COVID-19 testing capacity, particularly during periods of high virus transmission. To optimise resources, countries are encouraged to build upon their existing influenza surveillance infrastructure while aligning with WHO guidelines for SARS-CoV-2 surveillance.

The genomic sequencing data generated through the HBIS platform underscore the critical role of surveillance in monitoring SARS-CoV-2 variants. Since initiating genomic sequencing in April 2021, we have detected all major waves caused by different variants through our platform, including the emergence of the beta and delta variants in April and May 2021, respectively, followed by omicron subvariants from 2022 to 2024. These findings align with the SARS-CoV-2 genomic sequencing data reported by the Government of Bangladesh.^21^^,^^24^ This surveillance platform continues to share sequence data with the MoHFW, Government of Bangladesh, thereby updating information on SARS-CoV-2 variants across the country.

Establishing a dedicated surveillance platform for SARS-CoV-2 is labour- and resource-intensive and may not be sustainable in the long term for Bangladesh and other resource-constrained settings. Since staff salaries for influenza sentinel surveillance were covered by surveillance funding, and no additional costs were incurred for sample collection logistics, transportation, or storage except for the sequencing of SARS-CoV-2 variants, integrating SARS-CoV-2 surveillance into existing influenza sentinel surveillance systems could optimise resource utilisation. This integration can support routine monitoring and provide early signals of increased SARS-CoV-2 activity, thereby aiding in the development of appropriate public health responses and decisions.

Our influenza surveillance platform effectively monitored both influenza and SARS-CoV-2 from 2020 to 2024, helping track changes in the causes of respiratory infections in Bangladesh. Systematic testing facilitated the detection of both viruses among enrolled patients and identified temporal changes in their respective contributions. SARS-CoV-2 was the predominant circulating virus in 2020 and 2021. In 2022, both influenza and SARS-CoV-2 circulated at similar rates, whereas influenza emerged as the dominant virus in 2023 and 2024. This platform provided critical data for SARS-CoV-2 monitoring, including virus circulation patterns, peak transmission times, infection rates, deaths among SARS-CoV-2-infected patients, and genomic sequencing insights. These data will enable the Government of Bangladesh to make informed public health decisions in future, particularly if the national COVID-19 platform is discontinued.

Beyond hospital-based surveillance systems like HBIS platform, alternative surveillance modalities such as wastewater-based surveillance and rapid antigen testing (RAT) networks have also been explored in Bangladesh. Wastewater surveillance, which has been used globally as an early indicator of SARS-CoV-2 activity, showed initial promise in Bangladesh for tracking community-level transmission trends.^29^^,^^30^ However, its widespread implementation faced significant infrastructural and operational challenges, limiting its utility beyond localised pilot studies.^29^^,^^30^ Similarly, RATs were introduced nationwide to expand decentralised testing and provide rapid results, particularly in travel hubs and symptomatic individuals. Despite their affordability and speed, RATs were constrained by lower sensitivity, reduced detection capacity among asymptomatic individuals, and limited data capture. In contrast, the HBIS platform provided comprehensive individual-level data, including clinical presentation, epidemiological context, and genomic sequencing results, which are critical for informed public health responses. While each modality has inherent strengths and limitations, an integrated surveillance approach like the HBIS platform may offer a more reliable and cost-effective way to monitor SARS-CoV-2 in settings with limited resources.

There are several strengths in this study. First, it is one of the few large-scale, multi-year, multicentre prospective surveillance studies conducted in a low-resource setting to examine whether an existing influenza sentinel surveillance platform could be leveraged for SARS-CoV-2 monitoring. The HBIS platform enabled consistent, year-round data collection across geographically diverse sites, capturing both severe (SARI) and mild (ILI) respiratory illnesses. Second, the use of standardised WHO case definitions for SARI and ILI, systematic clinical data collection, and multiplex rRT-PCR testing ensured high-quality and comparable data for both influenza and SARS-CoV-2. Third, the integration of genomic sequencing enabled real-time tracking of circulating SARS-CoV-2 variants, supporting both national and global variant surveillance efforts. Importantly, the platform demonstrated that SARS-CoV-2 positivity trends in HBIS platform closely aligned with those in the national COVID-19 platform, despite differences in case definitions and testing strategies, highlighting its reliability and scalability. Overall, this study presents a practical and cost-effective model for integrated respiratory virus surveillance in resource-limited settings, aligned with WHO recommendations for integrated sentinel surveillance and pandemic preparedness.

However, our study has several limitations that should be considered when interpreting the findings. First, SARS-CoV-2 has been shown to cause a wide range of clinical manifestations, including gastrointestinal, neurological, cardiovascular, and asymptomatic presentations, many of which are not captured by the HBIS platform. As HBIS platform only detected medically attended illnesses, the SARS-CoV-2 positivity rate observed in HBIS platform may not accurately reflect the true positivity rate or SARS-CoV-2-related mortality rate reported by the national COVID-19 platform. As evidenced by Bangladesh's national COVID-19 platform, a large number of asymptomatic persons who did not meet the SARI and ILI case definitions were tested for SARS-CoV-2 for travel or administrative purposes rather than for medical reasons. Second, we were unable to conduct detailed comparative analyses between the HBIS platform and the national COVID-19 platform due to major gaps in the publicly available national COVID-19 data. The national platform lacked information on essential socio-demographic factors, such as age, sex, residence, occupation, and travel history for each SARS-CoV-2–confirmed case, which were available in HBIS platform. More importantly, clinical and epidemiological variables, including presenting symptoms, comorbidities, and treatment interventions, were comprehensively recorded in HBIS but were absent from the national platform. Additionally, laboratory findings related to other respiratory viruses and detailed clinical outcomes were also unavailable in the national COVID-19 platform. This lack of harmonised and granular data severely limited our ability to perform direct, variable-by-variable comparisons, hindering in-depth assessments of clinical profiles, healthcare utilisation, and outcomes across the two surveillance systems. Third, the HBIS platform experienced a substantial decline in the enrolment of patients with SARI in 2020. This reduction likely resulted from decreased health-seeking behaviour due to fear of contracting COVID-19 in hospitals and logistical challenges in maintaining routine surveillance operations.

Fourth, the correlation analysis comparing SARS-CoV-2 positivity trends between the HBIS and national COVID-19 platforms did not account for differences in testing criteria and population characteristics between the two platforms. Due to the lack of individual-level data in the publicly available COVID-19 platform dataset, we were unable to apply stratified or time-series modelling to adjust for these differences. Fifth, the HBIS surveillance system operated solely at tertiary care hospitals, limiting its representation in hard-to-reach areas and effectiveness in timely outbreak detection. Expanding the sentinel surveillance network to include rural and underserved regions, along with integrating data from other health systems, could provide a more comprehensive understanding of SARS-CoV-2 circulation in the country.

In Bangladesh, SARS-CoV-2 circulated from 2020 to 2024, with distinct peaks across epidemiological weeks and the detection of multiple variants. The trend of SARS-CoV-2 detection from HBIS platform aligned with national COVID-19 platform, demonstrating its potential as a sustainable platform for COVID-19 monitoring. Our findings underscore that influenza sentinel surveillance could serve as an early warning system for future COVID-19 outbreaks or other respiratory viruses of pandemic concern in Bangladesh and similar settings.

## Contributors

Study conceptualisation: MAI, MZH, FC; Methodology: MAI, MZH,SB, MAA, ZA, FC; Software: MAI, SB; Investigation: MAI; FC; Resources: MAI, SB; Validation: MAI, SB, PKG; Formal analysis: MAI, SB; Data curation: MAI, SB; Original draft preparation: MAI, MZH, SB, FC; Supervision: MAI,MZH, FC; Funding acquisition: MAI, FC; Visualisation: MAI, SB; Project administration: MAI, MZH, FC; Writing-review and editing: MAI, MZH, ZA, SB,TAS, MAA, PKG, A, MAAJB, MR, MJ, MNB, YK, MZR, MEH, SNMRA, ANA, MFRS, M, MHKJ, MR, TS, MR, FC; All authors had access to the data and critically reviewed the manuscript for important intellectual content and approved the final version. MAI, and FC had the final responsibility to submit for publication.

## Data sharing statement

In accordance with the data policies of the contributing institutions, and to protect intellectual property rights, the primary dataset (HBIS platform) cannot be made publicly available by the authors. However, it may be made available upon reasonable request to the Institutional Data Access Committees of the contributing institutions. Data from the national COVID-19 platform are available on the government dashboard and can be accessed at: Directorate General of Health Services, Government of Bangladesh. COVID-19 Dynamic Dashboard for Bangladesh. Dhaka, Bangladesh; 2025. Available from: https://dashboard.dghs.gov.bd/pages/covid19.php. Genomic sequencing data are available to registered users through the GISAID platform (https://www.gisaid.org), in accordance with GISAID's terms of use, using the IDs provided in Supplementary Table S5.

### Editor note

The Lancet Group takes a neutral position with respect to territorial claims in published maps and institutional affiliations.

## Declaration of generative AI and AI-assisted technologies in the writing process

During the preparation of this work, we used Grammarly AI to assist with grammar checking and language refinement. After using this tool, we thoroughly reviewed and edited the content as needed and take full responsibility for the final content of the publication.

## Declaration of interests

We declare no competing interests.
