# Supplementary material for: Leveraging the influenza sentinel surveillance platform for SARS-CoV-2 monitoring in Bangladesh (2020–2024): a prospective sentinel surveillance study
Source: Lancet Reg Health Southeast Asia. 2025 Aug 21;41:100657. doi: 10.1016/j.lansea.2025.100657 (PMC12398867; doi:10.1016/j.lansea.2025.100657)
Supplement: Supplementary Tables [file mmc1.docx]

**Table S1:** Availability of data regarding socio-demographical, clinical, and laboratory aspects in both HBIS and national COVID platforms in Bangladesh, March 2020– December 2024

| **Characteristic** | **Availability of data in both surveillance systems** | |
| --- | --- | --- |
| **Demographic Information** | **HBIS** | **National COVID-19** |
| Age | Yes | Yes (only for deceased patient) |
| Sex | Yes | Yes (only for deceased patient) |
| Residential address | Yes | Yes (only for deceased patient) |
| Occupation | Yes | Not available |
| Travel history | Yes | Not available |
| **Clinical Characteristics** |  |  |
| Fever | Yes | Not available |
| Cough | Yes | Not available |
| Runny nose | Yes | Not available |
| Headache (patients aged ≥5 years) | Yes | Not available |
| Sore throat | Yes | Not available |
| Difficulty breathing reported on admission | Yes | Not available |
| Chest indrawing (children aged <5 years) | Yes | Not available |
| Stridor in a calm child (children aged <5 years) | Yes | Not available |
| Being unable to drink (children aged <5 years) | Yes | Not available |
| Lethargy or unconsciousness (children aged <5 years) | Yes | Not available |
| Vomits everything (children aged <5 years) | Yes | Not available |
| History of convulsions (children aged <5 years) | Yes | Not available |
| Body ache | Yes | Not available |
| Diarrhea | Yes | Not available |
| Smoking history | Yes | Not available |
| Pregnancy history | Yes | Not available |
| Mental status of the patients | Yes | Not available |
| Duration of symptoms prior to admission in days (SARI) | Yes | Not available |
| Length of hospital stay in days (SARI) | Yes | Not available |
| **Comorbidity** |  |  |
| COPD | Yes | Not available |
| Asthma | Yes | Not available |
| Diabetes | Yes | Not available |
| Heart diseases | Yes | Not available |
| Hypertension | Yes | Not available |
| Cancer | Yes | Not available |
| Liver disease | Yes | Not available |
| Kidney disease | Yes | Not available |
| **Treatment received** |  |  |
| Antibiotic | Yes | Not available |
| Oseltamivir | Yes | Not available |
| Oxygen | Yes | Not available |
| Mechanical ventilation | Yes | Not available |
| ICU support | Yes | Not available |
| **Influenza and SARS-CoV-2 testing findings** |  |  |
| SARS-CoV-2 detected | Yes | Yes |
| Influenza virus detected | Yes | Not available |
| Co-infection (SARS-CoV-2 detected and influenza) | Yes | Not available |
| Influenza types | Yes | Not available |
| Influenza A | Yes | Not available |
| Influenza B | Yes | Not available |
| Influenza A and B | Yes | Not available |
| **Clinical Outcome** |  |  |
| Number of deaths | Yes | Yes |
| Place of death | Yes | Not available |

HBIS: Hospital based influenza sentinel surveillance in Bangladesh

**Table S2:** Comparison of clinical characteristics and outcomes among patients with SARS-CoV-2 and influenza in hospital-based influenza sentinel surveillance in Bangladesh, March 2020–December 2024

| **Characteristics** | **SARS-CoV-2 N=1,680; n (%)** | **Influenza  N=3,301; n (%)** | **Co-infected^a^ N=43; n (%)** | **P-value** |
| --- | --- | --- | --- | --- |
| Median age (years); Median (IQR) | 42 (23.5-60) | 16 (3.5-35) | 26 (9-55) | <0.001 |
| Sex (male) | 1,014 (60.4) | 1,923 (58.3) | 28 (65.1) | 0.259 |
| Fever | 1,680 (100) | 3,301 (100) | 43 (100) | - |
| Cough | 1,680 (100) | 3,301 (100) | 43 (100) | - |
| Difficulty breathing | 1,037 (82.6) | 847 (76.9) | 21 (87.5) | 0.002 |
| Co-morbid condition (≥ 1) | 578 (34.4) | 456 (13.8) | 10 (23.3) | <0.001 |
| **Patients aged<5 years** | **N=226** | **N=941** | **N=9** |  |
| Chest indrawing | 112 (55.5) | 252 (31.2) | 2 (22.2) | <0.001 |
| Stridor | 26 (12.9) | 48 (6.0) | 0 (0) | 0.002 |
| Unable to drink | 53 (26.2) | 153 (19.0) | 0 (0) | 0.023 |
| Vomit | 4 (4.0) | 52 (6.4) | 1 (11) | 0.333 |
| Lethargy | 2 (1.0) | 2 (0.3) | 0 (0) | 0.316 |
| Runny nose | 158 (69.9) | 747 (79.4) | 6 (66.7) | 0.007 |
| **Patients aged ≥5 years** | **N=1,454** | **N=2,360** | **N=34** |  |
| Body ache | 748 (51.4) | 1,124 (47.6) | 16 (47.1) | 0.071 |
| Headache | 680 (46.8) | 1,145 (48.5) | 15 (44.1) | 0.524 |
| Sore throat | 310 (25.2) | 439 (47.5) | 4 (22.2) | <0.001 |
| **Hospitalization Characteristics** | **N=1,680** | **N=3,301** | **N=43** |  |
| Duration of symptoms prior to admission in days^b^; Median (IQR) | 5 (3-7) | 4 (3-5) | 5 (3-7) | <0.001 |
| Length of hospital stay in days^c^; Median (IQR) | 3 (2-6) | 3 (2-4) | 3 (2-4) | <0.001 |
| Oxygen received | 968 (64.2) | 653 (26.0) | 12 (31.6) | <0.001 |
| Mechanical ventilation support | 2 (0.1) | 0 (0) | 0 (0) | - |
| ICU support | 4 (0.3) | 3 (0.1) | 0 (0) | - |
| **Clinical Outcome** | **N=1,507** | **N=2,514** | **N=38** |  |
| In-hospital death^d^ | 136 (9.02) | 24 (0.95) | 0 (0) | <0.001 |

^a^Co-infected with influenza and SARS-CoV-2

^b^Data on the duration of symptoms prior to admission are available for patients with severe acute respiratory infection

^c^Data on the length of hospital stay are available for patients with severe acute respiratory infection

^d^Data on deaths are available for patients with severe acute respiratory infection

**Table S3:** Comparison of SARS-CoV-2 positivity between national COVID-19 platform in Bangladesh and HBIS platform, March 2020– December 2024

| **Epi week** | **SARS-CoV-2 positivity** | | **Difference of positivity** | **95% Confidence Interval** | |
| --- | --- | --- | --- | --- | --- |
|  | **National COVID-19** | **HBIS** |  | **Lower value** | **Upper value** |
| 2020-10 | 6% | 0% | — | — | — |
| 2020-11 | 2% | 0% | — | — | — |
| 2020-12* | 20% | 0% | — | — | — |
| 2020-13 | 5% | 0% | — | — | — |
| 2020-14 | 2% | 0% | — | — | — |
| 2020-15 | 6% | 0% | — | — | — |
| 2020-16* | 13% | 0% | — | — | — |
| 2020-17 | 13% | 5% | 8% | -0.2% | 17.2% |
| 2020-18 | 12% | 4% | 8% | -0.7% | 15.0% |
| 2020-19 | 12% | 19% | -7% | -23.6% | 9.9% |
| 2020-20 | 14% | 18% | -4% | -17.0% | 9.3% |
| 2020-21* | 16% | 0% | — | — | — |
| 2020-22 | 20% | 24% | -4% | -21.9% | 14.5% |
| 2020-23* | 21% | 35% | -14% | -31.9% | 4.6% |
| 2020-24 | 20% | 19% | 1% | 10.1% | 12.4% |
| 2020-25 | 22% | 21% | 1% | -14.4% | 16.0% |
| 2020-26* | 22% | 33% | -11% | -23.3% | 1.5% |
| 2020-27 | 21% | 28% | -7% | -18.3% | 5.6% |
| 2020-28 | 22% | 28% | -6% | -16.0% | 5.0% |
| 2020-29* | 24% | 37% | -13% | -26.5% | 0.5% |
| 2020-30 | 23% | 29% | -6% | -16.3% | 4.4% |
| 2020-31 | 22% | 21% | 1% | -10.9% | 13.4% |
| 2020-32 | 25% | 27% | -2% | -18.6% | 11.8% |
| 2020-33* | 21% | 33% | -12% | -25.2% | 0.7% |
| 2020-34 | 20% | 23% | -3% | -12.8% | 6.1% |
| 2020-35* | 17% | 37% | -20% | -31.5% | -7.1% |
| 2020-36 | 16% | 13% | 3% | -3.9% | 10.6% |
| 2020-37 | 13% | 13% | 0% | -7.1% | 6.6% |
| 2020-38 | 12% | 8% | 4% | -2.6% | 10.8% |
| 2020-39 | 12% | 8% | 4% | -1.0% | 9.2% |
| 2020-40 | 12% | 9% | 3% | -4.9% | 11.4% |
| 2020-41 | 11% | 3% | 8% | 4.5% | 12.0% |
| 2020-42 | 11% | 4% | 7% | 2.8% | 11.8% |
| 2020-43 | 11% | 5% | 6% | 0.2% | 10.5% |
| 2020-44 | 11% | 2% | 9% | 4.9% | 13.4% |
| 2020-45 | 12% | 7% | 5% | -2.5% | 11.4% |
| 2020-46 | 12% | 4% | 8% | 3.1% | 13.4% |
| 2020-47 | 14% | 5% | 9% | -0.2% | 18.0% |
| 2020-48* | 14% | 24% | -10% | -24.0% | 3.6% |
| 2020-49 | 14% | 7% | 7% | 1.4% | 14.0% |
| 2020-50 | 13% | 9% | 4% | -4.8% | 11.9% |
| 2020-51* | 10% | 0% | — | — | — |
| 2020-52 | 9% | 14% | -5% | -19.2% | 9.5% |
| 2020-53 | 8% | 4% | 4% | 0.8% | 7.2% |
| 2021-1 | 7% | 3% | 4% | 0.5% | 6.8% |
| 2021-2 | 6% | 7% | -1% | 36.0% | 48.0% |
| 2021-3* | 4% | 20% | -16% | -28.9% | -2.4% |
| 2021-4 | 3% | 0% | — | — | — |
| 2021-5 | 3% | 9% | -6% | -12.7% | 1.0% |
| 2021-6 | 3% | 3% | 0% | -5.8% | 5.2% |
| 2021-7 | 3% | 0% | — | — | — |
| 2021-8 | 3% | 6% | -3% | -12.2% | 5.1% |
| 2021-9 | 4% | 7% | -3% | -9.9% | 2.7% |
| 2021-10* | 6% | 22% | -16% | -30.2% | -3.0% |
| 2021-11 | 9% | 8% | 1% | -9.0% | 11.5% |
| 2021-12 | 13% | 22% | -9% | -21.9% | 4.6% |
| 2021-13 | 21% | 25% | -4% | -15.3% | 6.6% |
| 2021-14 | 22% | 21% | 1% | -5.2% | 8.1% |
| 2021-15 | 21% | 29% | -8% | -18.2% | 0.7% |
| 2021-16 | 16% | 24% | -8% | -16.0% | -0.3% |
| 2021-17* | 11% | 25% | -14% | -24.1% | -2.5% |
| 2021-18 | 9% | 12% | -3% | -8.5% | 2.7% |
| 2021-19* | 9% | 22% | -13% | -29.9% | 3.8% |
| 2021-20 | 8% | 16% | -8% | -15.2% | -1.9% |
| 2021-21* | 9% | 22% | -13% | -21.0% | -4.9% |
| 2021-22* | 10% | 28% | -18% | -24.3% | -11.2% |
| 2021-23* | 12% | 30% | -18% | -25.4% | -9.7% |
| 2021-24* | 16% | 27% | -11% | -18.3% | -4.8% |
| 2021-25 | 20% | 26% | -6% | -24.4% | -2.6% |
| 2021-26 | 25% | 34% | -9% | -17.4% | -0.1% |
| 2021-27 | 31% | 36% | -5% | -15.5% | 6.0% |
| 2021-28* | 29% | 47% | -18% | -25.7% | -10.1% |
| 2021-29* | 31% | 46% | -15% | -35.6% | 4.2% |
| 2021-30* | 30% | 48% | -18% | -26.0% | -9.6% |
| 2021-31 | 28% | 31% | -3% | -10.7% | 5.9% |
| 2021-32 | 23% | 28% | -5% | -12.4% | 2.1% |
| 2021-33 | 19% | 17% | 2% | -6.0% | 9.4% |
| 2021-34 | 15% | 10% | 5% | -0.6% | 9.7% |
| 2021-35 | 11% | 11% | 0% | -6.5% | 7.6% |
| 2021-36 | 9% | 5% | 4% | 0.8% | 7.2% |
| 2021-37 | 7% | 4% | 3% | -1.0% | 5.4% |
| 2021-38 | 5% | 3% | 2% | -0.1% | 4.8% |
| 2021-39 | 4% | 1% | 3% | 1.9% | 4.6% |
| 2021-40 | 3% | 5% | -2% | -7.2% | 3.3% |
| 2021-41 | 2% | 3% | -1% | -4.3% | 2.6% |
| 2021-42 | 2% | 1% | 1% | -2.0% | 3.0% |
| 2021-43 | 1% | 0% | — | — | — |
| 2021-44 | 1% | 0% | — | — | — |
| 2021-45 | 1% | 0% | — | — | — |
| 2021-46 | 1% | 2% | -1% | -4.4% | 3.0% |
| 2021-47 | 1% | 2% | -1% | -4.0% | 3.0% |
| 2021-48 | 1% | 4% | -3% | -7.0% | 2.6% |
| 2021-49 | 1% | 4% | -3% | -7.8% | 2.7% |
| 2021-50 | 1% | 0% | — | — | — |
| 2021-51 | 2% | 0% | — | — | — |
| 2021-52 | 2% | 3% | -1% | -6.3% | 4.9% |
| 2022-1 | 4% | 0% | — |  |  |
| 2022-2 | 11% | 8% | 2.6% | -4.4% | 9.7% |
| 2022-3 | 25% | 28% | -3.0% | -15.0% | 8.8% |
| 2022-4 | 32% | 28% | 4.4% | -7.0% | 16.0% |
| 2022-5* | 27% | 48% | -21.5% | -33.7% | -9.2% |
| 2022-6* | 19% | 38% | -18.7% | -30.5% | -6.8% |
| 2022-7* | 12% | 24% | -11.6% | -22.9% | -0.4% |
| 2022-8 | 6% | 9% | -3.2% | -13.3% | 6.8% |
| 2022-9* | 3% | 17% | -14.0% | -23.7% | -4.3% |
| 2022-10 | 2% | 2% | -0.1% | -4.3% | 4.2% |
| 2022-11 | 2% | 0% | 0.0% | 0.0% | 0.0% |
| 2022-12 | 1% | 2% | -1.2% | -5.5% | 3.1% |
| 2022-13 | 1% | 0% | 0.0% | 0.0% | 0.0% |
| 2022-14 | 1% | 4% | -2.9% | -7.9% | 2.0% |
| 2022-15 | 1% | 2% | -1.8% | -6.4% | 2.9% |
| 2022-16 | 1% | 0% | — | — | — |
| 2022-17 | 0% | 0% | — | — | — |
| 2022-18 | 1% | 0% | — | — | — |
| 2022-19 | 1% | 0% | — | — | — |
| 2022-20 | 1% | 0% | — | — | — |
| 2022-21 | 1% | 0% | — | — | — |
| 2022-22 | 1% | 0% | — | — | — |
| 2022-23 | 1% | 0% | — | — | — |
| 2022-24 | 4% | 0% | — | — | — |
| 2022-25 | 12% | 5% | 6.7% | 3.0% | 10.3% |
| 2022-26* | 15% | 4% | 11.5% | 8.6% | 14.4% |
| 2022-27 | 16% | 10% | 6.3% | 1.6% | 10.9% |
| 2022-28 | 13% | 14% | -90.0% | -8.3% | 6.5% |
| 2022-29 | 10% | 8% | 2.3% | -1.4% | 6.0% |
| 2022-30 | 7% | 9% | -2.4% | -6.9% | 2.1% |
| 2022-31 | 6% | 8% | -1.7% | -6.6% | 3.2% |
| 2022-32 | 5% | 4% | 1.2% | -2.2% | 4.6% |
| 2022-33 | 4% | 7% | -2.7% | -8.0% | 2.5% |
| 2022-34 | 4% | 5% | -0.6% | -4.2% | 2.9% |
| 2022-35 | 5% | 5% | 0.4% | -3.2% | 4.0% |
| 2022-36 | 7% | 4% | 3.3% | 0.1% | 6.4% |
| 2022-37 | 9% | 1% | 7.9% | 5.8% | 10.0% |
| 2022-38 | 13% | 15% | -1.6% | -8.2% | 5.0% |
| 2022-39 | 14% | 14% | 0.0% | -5.4% | 5.3% |
| 2022-40 | 12% | 9% | 3.6% | -4.7% | 11.9% |
| 2022-41 | 9% | 10% | -1.6% | -9.4% | 6.3% |
| 2022-42 | 6% | 8% | -1.5% | -8.9% | 5.9% |
| 2022-43 | 4% | 2% | 2.1% | -1.7% | 6.0% |
| 2022-44 | 3% | 0% | — | — | — |
| 2022-45 | 2% | 0% | — | — | — |
| 2022-46 | 1% | 0% | — | — | — |
| 2022-47 | 1% | 0% | — | — | — |
| 2022-48 | 1% | 0% | — | — | — |
| 2022-49 | 1% | 0% | — | — | — |
| 2022-50 | 1% | 0% | — | — | — |
| 2022-51 | 1% | 1% | -0.1% | -1.7% | 1.5% |
| 2022-52 | 1% | 0% | — | — | — |
| 2023-1 | 1% | 1% | 0% | -2.4% | 1.6% |
| 2023-2 | 1% | 0% | — | — | — |
| 2023-3 | 0% | 0% | — | — | — |
| 2023-4 | 1% | 0% | — | — | — |
| 2023-5 | 1% | 0% | — | — | — |
| 2023-6 | 0% | 0% | — | — | — |
| 2023-7 | 1% | 0% | — | — | — |
| 2023-8 | 0% | 0% | — | — | — |
| 2023-9 | 0% | 0% | — | — | — |
| 2023-10 | 0% | 0% | — | — | — |
| 2023-11 | 0% | 0% | — | — | — |
| 2023-12 | 0% | 0% | — | — | — |
| 2023-13 | 0% | 0% | — | — | — |
| 2023-14 | 0% | 0% | — | — | — |
| 2023-15 | 1% | 0% | — | — | — |
| 2023-16 | 1% | 0% | — | — | — |
| 2023-17 | 1% | 1% | 0% | -1.6% | 1.7% |
| 2023-18 | 1% | 0% | — | — | — |
| 2023-19 | 2% | 1% | 1% | -0.6% | 2.0% |
| 2023-20 | 2% | 3% | -1% | -3.6% | 1.2% |
| 2023-21 | 4% | 1% | 3% | 2.5% | 5.0% |
| 2023-22 | 6% | 7% | -1% | -4.6% | 3.0% |
| 2023-23 | 7% | 7% | 0% | -4.1% | 3.4% |
| 2023-24 | 7% | 6% | 1% | -2.8% | 4.6% |
| 2023-25 | 8% | 14% | -6% | -12.0% | -1.5% |
| 2023-26 | 7% | 7% | 0% | -8.5% | 7.5% |
| 2023-27 | 5% | 10% | -5% | -9.7% | -0.6% |
| 2023-28 | 4% | 8% | -4% | -6.8% | 0.4% |
| 2023-29 | 4% | 12% | -8% | -11.6% | -3.3% |
| 2023-30 | 4% | 5% | -1% | -4.9% | 2.5% |
| 2023-31 | 3% | 5% | -2% | -3.8% | 2.7% |
| 2023-32 | 2% | 1% | 1% | -0.5% | 2.7% |
| 2023-33 | 2% | 1% | 1% | -0.6% | 2.3% |
| 2023-34 | 2% | 1% | 1% | -1.3% | 1.9% |
| 2023-35 | 2% | 1% | 1% | 0.0% | 2.4% |
| 2023-36 | 1% | 1% | 0% | -0.2% | 1.7% |
| 2023-37 | 1% | 0% | — | — | — |
| 2023-38 | 1% | 1% | 0% | -1.2% | 1.8% |
| 2023-39 | 1% | 0% | — | — | — |
| 2023-40 | 1% | 0% | — | — | — |
| 2023-41 | 1% | 2% | -1% | -4.0% | 2.8% |
| 2023-42 | 1% | 0% | — | — | — |
| 2023-43 | 1% | 0% | — | — | — |
| 2023-44 | 1% | 0% | — | — | — |
| 2023-45 | 1% | 0% | — | — | — |
| 2023-46 | 1% | 0% | — | — | — |
| 2023-47 | 1% | 0% | — | — | — |
| 2023-48 | 1% | 0% | — | — | — |
| 2023-49 | 1% | 0% | — | — | — |
| 2023-50 | 1% | 1% | 0% | -2.0% | 2.1% |
| 2023-51 | 1% | 2% | -1% | -3.4% | 1.8% |
| 2023-52 | 2% | 1% | 1% | -0.9% | 3.2% |
| 2024-1 | 6% | 2% | 4% | -0.4% | 8.4% |
| 2024-2 | 5% | 9% | -4% | -10.3% | 3.5% |
| 2024-3 | 6% | 3% | 3% | -1.9% | 7.5% |
| 2024-4 | 5% | 7% | -2% | -8.5% | 3.2% |
| 2024-5 | 7% | 6% | 1% | -4.0% | 5.0% |
| 2024-6 | 6% | 11% | -5% | -11.3% | 1.3% |
| 2024-7 | 9% | 12% | -3% | -9.3% | 3.3% |
| 2024-8 | 9% | 3% | 6% | 1.4% | 10.6% |
| 2024-9 | 7% | 6% | 1% | -3.9% | 4.9% |
| 2024-10 | 6% | 7% | -1% | -5.4% | 4.2% |
| 2024-11 | 6% | 5% | 1% | -3.4% | 4.8% |
| 2024-12 | 6% | 3% | 3% | -0.4% | 6.2% |
| 2024-13 | 5% | 3% | 2% | -0.3% | 5.9% |
| 2024-14 | 5% | 1% | 4% | 1.8% | 5.7% |
| 2024-15 | 4% | 0% | — | — | — |
| 2024-16 | 4% | 1% | 3% | 2.2% | 4.9% |
| 2024-17 | 4% | 1% | 3% | 1.6% | 4.8% |
| 2024-18 | 4% | 2% | 2% | 0.4% | 4.3% |
| 2024-19 | 4% | 2% | 2% | -0.4% | 3.9% |
| 2024-20 | 4% | 2% | 2% | 0.2% | 4.2% |
| 2024-21 | 5% | 4% | 1% | -2.3% | 3.7% |
| 2024-22 | 5% | 3% | 2% | -0.8% | 4.4% |
| 2024-23 | 5% | 7% | -2% | -6.2% | 1.3% |
| 2024-24 | 5% | 8% | -3% | -7.1% | 1.2% |
| 2024-25 | 5% | 9% | -4% | -11.7% | 2.6% |
| 2024-26 | 5% | 8% | -3% | -6.6% | 0.6% |
| 2024-27 | 5% | 4% | 1% | -2.4% | 3.6% |
| 2024-28 | 5% | 5% | 0% | -2.7% | 3.5% |
| 2024-29 | 5% | 5% | 0% | -4.0% | 5.0% |
| 2024-30 | 4% | 6% | -2% | -8.1% | 4.3% |
| 2024-31 | 4% | 4% | 0% | -2.2% | 3.9% |
| 2024-32 | 4% | 2% | 2% | -1.6% | 5.5% |
| 2024-33 | 4% | 1% | 3% | -0.2% | 5.6% |
| 2024-34 | 4% | 1% | 3% | 0.1% | 6.6% |
| 2024-35 | 6% | 1% | 5% | 1.4% | 8.7% |
| 2024-36 | 4% | 0% | — | — | — |
| 2024-37 | 5% | 0% | — | — | — |
| 2024-38 | 5% | 1% | 4% | 0.9% | 8.0% |
| 2024-39 | 4% | 1% | 3% | 0.5% | 6.2% |
| 2024-40 | 5% | 2% | 3% | -0.7% | 7.7% |
| 2024-41 | 6% | 4% | 2% | -4.0% | 7.1% |
| 2024-42 | 5% | 2% | 3% | -1.4% | 6.3% |
| 2024-43 | 4% | 2% | 2% | -1.3% | 6.7% |
| 2024-44 | 7% | 5% | 2% | -3.5% | 7.8% |
| 2024-45 | 7% | 1% | 6% | 1.4% | 10.0% |
| 2024-46 | 5% | 1% | 4% | 0.4% | 8.9% |
| 2024-47 | 6% | 3% | 3% | -1.5% | 7.4% |
| 2024-48 | 4% | 1% | 3% | 0.0% | 7.0% |
| 2024-49 | 4% | 1% | 3% | -0.4% | 6.5% |
| 2024-50 | 6% | 1% | 5% | 0.7% | 9.1% |
| 2024-51 | 6% | 0% | — | — | — |
| 2024-52 | 5% | 0% | — | — | — |

*p ≤0.05, Chi-squared or Fisher’s exact test, as appropriate

HBIS: Hospital-based influenza sentinel surveillance in Bangladesh

**Table S4.** Incremental costs associated with the integration of SARS-CoV-2 monitoring into the hospital-based influenza sentinel surveillance platform, Bangladesh, March 2020–December 2024

| **Cost Category** | **Cost Coverage / Unit Cost** | **Additional Cost for SARS-CoV-2** |
| --- | --- | --- |
| Staff salaries | Covered by HBIS | No additional cost required |
| Operational expenses | Covered by HBIS | No additional cost required |
| Sample collection logistics, transportation and storage | Covered by HBIS | No additional cost required |
| **Sample testing (March 2020–October 2021)** | | |
| Solely Influenza testing (rRT-PCR^a^) | Covered by HBIS (~$30/sample^b^) | – |
| Solely SARS-CoV-2 testing (rRT-PCR^a^) | – | Additional cost of ~$30 per test^b^  (cost supported by HBIS) |
| **Sample testing (November 2021–December 2024)** | | |
| Influenza & SARS-CoV-2 multiplex testing (Flu SC2 assay) (rRT-PCR^a^) | Covered by HBIS (~$30/sample^b^) | No additional cost for SARS-CoV-2 testing |
| Whole-genome sequencing of SARS-CoV-2 | – | Additional cost of ~$80 per sequencing  (cost supported by HBIS) |

^a^rRT-PCR: Real-time reverse transcription polymerase chain reaction

^b^Estimated cost for laboratory testing; actual cost varied depending on batch size.

**Table S5**: GISAID accession identifiers of SARS-CoV-2 genomic sequences used in hospital-based sentinel influenza surveillance in Bangladesh, 2021–2024

| **Serial Number** | | **GISAID**  **ID** | **Serial Number** | **GISAID**  **ID** | **Serial Number** | **GISAID**  **ID** |
| --- | --- | --- | --- | --- | --- | --- |
| 1 | EPI_ISL_2627368 | | 69 | EPI_ISL_11324515 | 137 | EPI_ISL_19783347 |
| 2 | EPI_ISL_8422261 | | 70 | EPI_ISL_8422173 | 138 | EPI_ISL_19783328 |
| 3 | EPI_ISL_8422266 | | 71 | EPI_ISL_8422244 | 139 | EPI_ISL_8422225 |
| 4 | EPI_ISL_8422147 | | 72 | EPI_ISL_2627388 | 140 | EPI_ISL_2627371 |
| 5 | EPI_ISL_2627369 | | 73 | EPI_ISL_2627365 | 141 | EPI_ISL_2627364 |
| 6 | EPI_ISL_2627363 | | 74 | EPI_ISL_2627360 | 142 | EPI_ISL_1828782 |
| 7 | EPI_ISL_1828780 | | 75 | EPI_ISL_2235495 | 143 | EPI_ISL_2235494 |
| 8 | EPI_ISL_2233363 | | 76 | EPI_ISL_2233362 | 144 | EPI_ISL_2627370 |
| 9 | EPI_ISL_2627387 | | 77 | EPI_ISL_2627395 | 145 | EPI_ISL_2627362 |
| 10 | EPI_ISL_2627366 | | 78 | EPI_ISL_2627367 | 146 | EPI_ISL_2627361 |
| 11 | EPI_ISL_18839353 | | 79 | EPI_ISL_18839346 | 147 | EPI_ISL_19174002 |
| 12 | EPI_ISL_18271334 | | 80 | EPI_ISL_18271333 | 148 | EPI_ISL_18271332 |
| 13 | EPI_ISL_18271331 | | 81 | EPI_ISL_18271330 | 149 | EPI_ISL_18271329 |
| 14 | EPI_ISL_18271328 | | 82 | EPI_ISL_18271327 | 150 | EPI_ISL_18271326 |
| 15 | EPI_ISL_18271325 | | 83 | EPI_ISL_18271324 | 151 | EPI_ISL_18271323 |
| 16 | EPI_ISL_18271322 | | 84 | EPI_ISL_18271321 | 152 | EPI_ISL_18271320 |
| 17 | EPI_ISL_18271318 | | 85 | EPI_ISL_18271317 | 153 | EPI_ISL_18271316 |
| 18 | EPI_ISL_18271315 | | 86 | EPI_ISL_18271314 | 154 | EPI_ISL_18271313 |
| 19 | EPI_ISL_18271312 | | 87 | EPI_ISL_18271311 | 155 | EPI_ISL_18271310 |
| 20 | EPI_ISL_18271309 | | 88 | EPI_ISL_18271308 | 156 | EPI_ISL_18271307 |
| 21 | EPI_ISL_18271306 | | 89 | EPI_ISL_18271305 | 157 | EPI_ISL_18271304 |
| 22 | EPI_ISL_18271303 | | 90 | EPI_ISL_18271302 | 158 | EPI_ISL_19173998 |
| 23 | EPI_ISL_19174020 | | 91 | EPI_ISL_18014057 | 159 | EPI_ISL_18014056 |
| 24 | EPI_ISL_18014055 | | 92 | EPI_ISL_18012761 | 160 | EPI_ISL_17973325 |
| 25 | EPI_ISL_17973324 | | 93 | EPI_ISL_17973323 | 161 | EPI_ISL_17973322 |
| 26 | EPI_ISL_17973321 | | 94 | EPI_ISL_17973320 | 162 | EPI_ISL_17973319 |
| 27 | EPI_ISL_17973318 | | 95 | EPI_ISL_17973317 | 163 | EPI_ISL_17973315 |
| 28 | EPI_ISL_17973313 | | 96 | EPI_ISL_17973312 | 164 | EPI_ISL_17973311 |
| 29 | EPI_ISL_17973310 | | 97 | EPI_ISL_17973309 | 165 | EPI_ISL_17973308 |
| 30 | EPI_ISL_17973307 | | 98 | EPI_ISL_17973306 | 166 | EPI_ISL_18560356 |
| 31 | EPI_ISL_18560354 | | 99 | EPI_ISL_17703749 | 167 | EPI_ISL_17703748 |
| 32 | EPI_ISL_18271301 | | 100 | EPI_ISL_18271300 | 168 | EPI_ISL_18271299 |
| 33 | EPI_ISL_18271298 | | 101 | EPI_ISL_18269257 | 169 | EPI_ISL_17699545 |
| 34 | EPI_ISL_17699544 | | 102 | EPI_ISL_17699551 | 170 | EPI_ISL_17699550 |
| 35 | EPI_ISL_17699549 | | 103 | EPI_ISL_17699548 | 171 | EPI_ISL_17699547 |
| 36 | EPI_ISL_17699546 | | 104 | EPI_ISL_17973314 | 172 | EPI_ISL_19174029 |
| 37 | EPI_ISL_19174028 | | 105 | EPI_ISL_19174027 | 173 | EPI_ISL_19174026 |
| 38 | EPI_ISL_19174025 | | 106 | EPI_ISL_19174024 | 174 | EPI_ISL_19174023 |
| 39 | EPI_ISL_19174022 | | 107 | EPI_ISL_19174021 | 175 | EPI_ISL_19174019 |
| 40 | EPI_ISL_19174018 | | 108 | EPI_ISL_19174017 | 176 | EPI_ISL_19174016 |
| 41 | EPI_ISL_19174015 | | 109 | EPI_ISL_19174014 | 177 | EPI_ISL_19174012 |
| 42 | EPI_ISL_19174011 | | 110 | EPI_ISL_19174010 | 178 | EPI_ISL_19174009 |
| 43 | EPI_ISL_19174008 | | 111 | EPI_ISL_19174007 | 179 | EPI_ISL_19174006 |
| 44 | EPI_ISL_19174005 | | 112 | EPI_ISL_19174004 | 180 | EPI_ISL_19174003 |
| 45 | EPI_ISL_19174001 | | 113 | EPI_ISL_19174000 | 181 | EPI_ISL_19173999 |
| 46 | EPI_ISL_19261208 | | 114 | EPI_ISL_19783348 | 182 | EPI_ISL_19783346 |
| 47 | EPI_ISL_19783345 | | 115 | EPI_ISL_19783344 | 183 | EPI_ISL_19783343 |
| 48 | EPI_ISL_19783342 | | 116 | EPI_ISL_19783341 | 184 | EPI_ISL_19783340 |
| 49 | EPI_ISL_19783339 | | 117 | EPI_ISL_19783338 | 185 | EPI_ISL_19783337 |
| 50 | EPI_ISL_19783336 | | 118 | EPI_ISL_19783335 | 186 | EPI_ISL_19783334 |
| 51 | EPI_ISL_19783333 | | 119 | EPI_ISL_19783332 | 187 | EPI_ISL_19783331 |
| 52 | EPI_ISL_19783330 | | 120 | EPI_ISL_19783329 | 188 | EPI_ISL_19783327 |
| 53 | EPI_ISL_19783326 | | 121 | EPI_ISL_19785581 | 189 | EPI_ISL_19785580 |
| 54 | EPI_ISL_19783360 | | 122 | EPI_ISL_19783359 | 190 | EPI_ISL_19783358 |
| 55 | EPI_ISL_19783357 | | 123 | EPI_ISL_19783356 | 191 | EPI_ISL_19783355 |
| 56 | EPI_ISL_19783354 | | 124 | EPI_ISL_19783353 | 192 | EPI_ISL_19783352 |
| 57 | EPI_ISL_19783351 | | 125 | EPI_ISL_19783350 | 193 | EPI_ISL_19783349 |
| 58 | EPI_ISL_19261210 | | 126 | EPI_ISL_19261209 | 194 | EPI_ISL_19261207 |
| 59 | EPI_ISL_19261206 | | 127 | EPI_ISL_19261193 | 195 | EPI_ISL_19261192 |
| 60 | EPI_ISL_19261191 | | 128 | EPI_ISL_19261190 | 196 | EPI_ISL_19785582 |
| 61 | EPI_ISL_19791521 | | 129 | EPI_ISL_19791522 | 197 | EPI_ISL_19791523 |
| 62 | EPI_ISL_19791524 | | 130 | EPI_ISL_19791525 | 198 | EPI_ISL_19791526 |
| 63 | EPI_ISL_19791527 | | 131 | EPI_ISL_19791528 | 199 | EPI_ISL_19791529 |
| 64 | EPI_ISL_19791530 | | 132 | EPI_ISL_19791531 | 200 | EPI_ISL_19791532 |
| 65 | EPI_ISL_19791533 | | 133 | EPI_ISL_19791534 | 201 | EPI_ISL_19791535 |
| 66 | EPI_ISL_19791536 | | 134 | EPI_ISL_19791537 | 202 | EPI_ISL_19791538 |
| 67 | EPI_ISL_19791539 | | 135 | EPI_ISL_19791540 | 203 | EPI_ISL_19791541 |
| 68 | EPI_ISL_19791542 | | 136 | EPI_ISL_2233361 | 204 | EPI_ISL_1828779 |

***Note****: These data can be accessed by registered users at* [*https://www.gisaid.org*](https://www.gisaid.org) *in accordance with GISAID's terms of use.*
